# Helical springs as a color indicator for determining chirality and enantiomeric excess

**DOI:** 10.1126/sciadv.abg5381

**Published:** 2021-06-30

**Authors:** Katsuhiro Maeda, Daisuke Hirose, Mai Nozaki, Yoichi Shimizu, Taro Mori, Kentaro Yamanaka, Koji Ogino, Tatsuya Nishimura, Tsuyoshi Taniguchi, Munetsugu Moro, Eiji Yashima

**Affiliations:** 1 Nano Life Science Institute (WPI-NanoLSI), Kanazawa University, Kakuma-machi, Kanazawa 920-1192, Japan.; 2 Graduate School of Natural Science and Technology, Kanazawa University, Kakuma-machi, Kanazawa 920-1192, Japan.; 3 Graduate School of Frontier Science Initiative, Kanazawa University, Kakuma-machi, Kanazawa 920-1192, Japan.; 4 Forensic Science Laboratory, Okayama Prefectural Police Headquarters, 1-3-2 Tonda-cho, Kita-ku, Okayama 700-0816, Japan.; 5 Department of Molecular and Macromolecular Chemistry, Graduate School of Engineering, Nagoya University, Chikusa-ku, Nagoya 464-8603, Japan.

## Abstract

Chirality plays a key role in the physiological system, because molecular functionalities may drastically alter due to a change in chirality. We report herein a unique color indicator with a static helicity memory, which exhibits visible color changes in response to the chirality of chiral amines. A difference of less than 2% in the enantiomeric excess (ee) values causes a change in the absorption that is visible to the naked eyes. This was further quantified by digital photography by converting to RGB values. This system relies on the change in the tunable helical pitch of the π-conjugated polymer backbone in specific solvents and allows rapid on-site monitoring of chirality of nonracemic amines, including drugs, and the simultaneous quantitative determination of their ee values.

## Teaser

Helical polymer–based molecular systems allow naked-eye determination of chirality and enantiomeric excess of chiral amines.

## INTRODUCTION

Chirality is an important aspect in living systems. For instance, a pair of enantiomers often exhibits totally different physiological activities depending on the homochirality of the biological molecule. Therefore, rapid and reliable methods for determining the chirality (configuration) and enantiomeric excess (ee) of chiral molecules, particularly chiral drugs, are highly demanded in the pharmaceutical industry (*1*, *2*). Chromatographic enantioseparation by high-performance liquid chromatography (HPLC) has mostly been used for this purpose because it allows the precise determination of ee for various nonracemic compounds produced by the state-of-the-art asymmetric catalysis and traditional optical resolution of racemates (*3*, *4*).

**Fig. 1:**
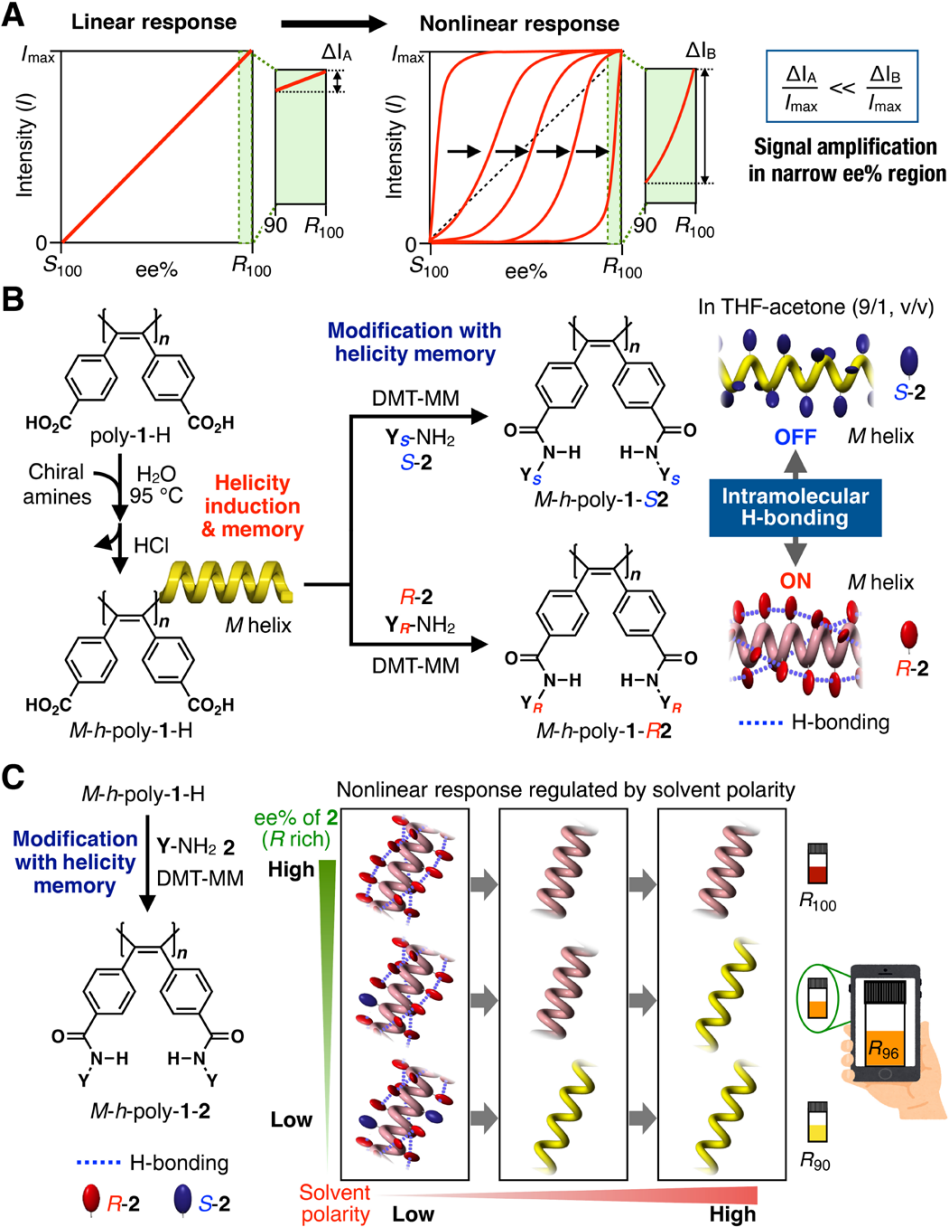
Helical polymer–based visual colorimetric sensor. (**A**) Schematic illustration of linear (left) and nonlinear (right) responses of output signals against ee of chiral guests. (**B**) Schematic illustration of the synthesis of left-handed helical *M*-*h*-poly-**1**-H with static helicity memory, followed by functionalization of the carboxy pendants with chiral amine **2**. Visual colorimetric detection of the absolute configuration of **2** due to change in the helical pitch of the chromophoric polymer backbone triggered by intramolecular H-bonding between the amide pendants (on/off switch). (**C**) Schematic illustration of functionalization of the carboxy pendants of *M*-*h*-poly-**1**-H with **2** and visual colorimetric determination of the ee of **2** relying on the nonlinear response of a change in the helical pitch of the chromophoric polymer backbone triggered by the on/off switch—solvent polarity–regulated formation of intramolecular H-bonding network among the amide pendants. The ee can be quantified by digital photography by converting to RGB values.

Another interesting class of techniques is based on enantioselective molecular probes/sensors (*5*–*12*). These methods provide only a rough estimation of the ee values, not as accurate as those determined by chiral HPLC. This is mostly because of the linear relationship between the full range of ee values and output signals, leading to unignorable deviations (errors) (Fig. 1A) (*13*, *14*). Anslyn and co-workers developed an elegant method for the accurate determination of ee values of certain chiral molecules in the high-ee region (*15*, *16*). The method was based on the use of covalent and noncovalent helical polymer systems that exhibited nonlinear circular dichroism (CD)–ee relationships (*10*, *17*–*19*). Such a nonlinear chiral response to ee has a substantial advantage over the linear chiral response, because chiral signals, particularly in the narrow, high, and/or low-ee regions of interest, can be remarkably amplified, thereby allowing the quantification of ee values with high accuracy (Fig. 1A).

Among the promising alternatives is the direct colorimetric discrimination between enantiomers and the simultaneous determination of ee values of the target chiral compounds, based on the differences in absorbance and fluorescence emission, thereby enabling the naked-eye detection of chirality. This is, however, challenging, and successful examples of these systems are limited due to the distinct conformational scaffold of low–molecular weight chiral receptors, which exhibit a linear response (*20*–*27*).

We recently reported that nonracemic amines in water induced the folding of π-conjugated fluorescent poly(diphenylacetylene) (PDPA) bearing carboxy pendants (poly-**1**-H) into a one-handed helix upon thermal annealing. The right (*P*)– or left (*M*)–handed helical conformation induced in poly-**1**-H by nonracemic amines could be retained, namely, “memorized,” after complete removal of the chiral amines, resulting in the formation of the one-handed helical *P*- or *M*-*h*-poly-**1**-H with static helicity memory, respectively (Fig. 1B) (*28*). As part of our ongoing program to develop *h*-poly-**1**-H–based advanced chiral materials for separating enantiomers (*29*), we serendipitously found that *M*-*h*-poly-**1**-Hs modified with (*S*)- and (*R*)-1-phenylethylamine (*S*- and *R*-**2a**), namely, *M*-*h*-poly-**1**-*S***2a** and *M*-*h*-poly-**1**-*R***2a**, respectively, showed completely different colors in specific solvents.

Here, we report an unprecedented helical polymer–based versatile color indicator that allows not only the assignment of the absolute configuration of chiral amines but also the quantitative determination of their ee values in the full range of ee. This was achieved by digital photography by converting to the RGB (red, green, and blue) values (Fig. 1, B and C). The method was as accurate as chiral HPLC. The present helical polymer–based color change relies on the nonlinear response of a change in the tunable helical pitch of the π-conjugated polymer backbone like a helical spring, which results from the formation or disruption (switching on/off) of the intramolecular hydrogen (H)–bonding networks among the pendant amides in specific polar solvent mixtures. This allows the rapid, on-site monitoring of the chirality of nonracemic amines and the simultaneous quantitative determination of their ee values. A rapid and simple system for the precise determination of ee of chiral amines, which are important components or precursors for pharmaceuticals and pesticides, would be particularly useful (*4*, *7*, *12*–*14*).

## RESULTS AND DISCUSSION

The one-handed helical *M*-*h*-poly-**1**-H and *P*-*h*-poly-**1**-H with static helicity memories were prepared from optically inactive poly-**1**-H based on the helicity induction and memory strategy that we developed previously (Fig. 1B and figs. S1 and S2) (*28*). The carboxy groups of *M*-*h*-poly-**1**-H were modified with *S*- and *R*-**2a** via amide linkage using 4-(4,6-dimethoxy-1,3,5-triazin-2-yl)-4-methylmorpholinium chloride (DMT-MM) as a condensing reagent, thereby quantitatively producing diastereomeric helices *M*-*h*-poly-**1**-*S***2a** and *M*-*h*-poly-**1**-*R***2a**, respectively (Fig. 1B and fig. S4) (*28*).

**Fig. 2:**
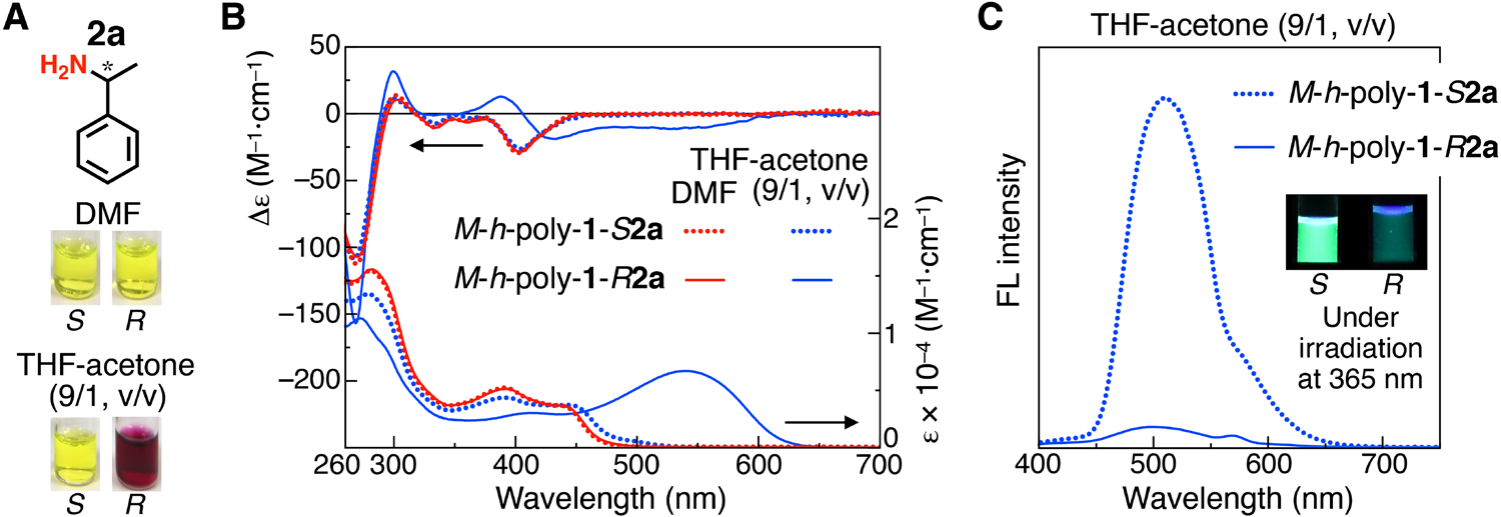
Naked-eye detection of enantiomers of amine 2a. (**A**) Color of 1.0 mM *M*-*h*-poly-**1**-*S***2a** and *M*-*h*-poly-**1**-*R***2a** in DMF and THF-acetone (9/1, v/v) at 25°C. (**B**) CD and absorption spectra of 1.0 mM *M*-*h*-poly-**1**-*S***2a** (dotted) and *M*-*h*-poly-**1**-*R***2a** (solid) in DMF (red) and THF-acetone (9/1, v/v) (blue) at 25°C. (**C**) Fluorescence spectra (excitation at 350 nm) of 0.01 mM *M*-*h*-poly-**1**-*S***2a** (dotted) and *M*-*h*-poly-**1**-*R***2a** (solid) in THF-acetone (9/1, v/v). Inset shows photographs under irradiation at 365 nm.

The solutions of *M*-*h*-poly-**1**-*S***2a** and *M*-*h*-poly-**1**-*R***2a** in *N*,*N*-dimethylformamide (DMF) were yellow in color and showed almost the same absorption and CD spectra (Fig. 2, A and B). These spectral patterns remained unchanged at 25°C for 24 hours and were similar to those of *M*-*h*-poly-**1**-H (fig. S8), suggesting that postmodification hardly affected the original helicity memory and helix-sense excess (*hse*) values even after introducing the enantiomeric amide pendant groups. When dissolved in tetrahydrofuran (THF)–acetone (9/1, v/v), the absorption spectrum of *M*-*h*-poly-**1**-*R***2a** notably red-shifted by ~100 nm and exhibited a remarkable hypochromic effect in the aromatic absorption region (260 to 300 nm). The CD spectrum also changed remarkably, and the solution color changed from yellow to deep red (Fig. 2, A and B). In contrast, there was only a slight change in the spectra of *M*-*h*-poly-**1**-*S***2a** in THF-acetone, and the solution color remained yellow, same as that in DMF. This striking color change enabled the naked-eye detection of the enantiomers of **2a** at 1.0 mM (Fig. 2A) as well as at 0.1 and 0.05 mM (fig. S10D). In a dilute solution (0.01 mM), the fluorescence emission of *M*-*h*-poly-**1**-*R***2a** was largely quenched, as anticipated from the hypochromic effect. The fluorescence quantum yields (Φ) for *M*-*h*-poly-**1**-*S***2a** and *M*-*h*-poly-**1**-*R***2a** in THF-acetone (9/1, v/v) were determined to be 30.9% and 8.0%, respectively, using quinine sulfate in aqueous sulfuric acid (0.1 M) as a standard material (*30*). Hence, the chirality of **2a** can also be visually discriminated by its fluorescence (Fig. 2C). The absorption and CD spectra of *M*-*h*-poly-**1**-*S***2a** and *M*-*h*-poly-**1**-*R***2a** in THF-acetone (9/1, v/v) and DMF were completely independent of the polymer concentration (0.025 to 2.5 mM; fig. S9), thus eliminating the possibility of the color change arising due to the formation of aggregates in THF-acetone (9/1, v/v).

As expected, the spectral behaviors of *P*-*h*-poly-**1**-*S***2a** and *P*-*h*-poly-**1**-*R***2a** prepared from the opposite right-handed helical *P*-*h*-poly-**1**-H were totally opposite to those of *M*-*h*-poly-**1**-*R***2a** and *M*-*h*-poly-**1**-*S***2a**, respectively, and mirror-image CD spectra were obtained in THF-acetone (9/1, v/v) (fig. S10). In addition, the colorimetric and spectral differences between *M*-*h*-poly-**1**-*S***2a** and *M*-*h*-poly-**1**-*R***2a** were highly dependent on the *hse* of *h*-poly-**1**-H and decreased with decreasing *hse* values of *h*-poly-**1**-H before its modification (fig. S11). The reactions of *h*-poly-**1**-H with *R*- and *S*-**2a** using DMT-MM proceeded very quickly; the reaction reached completion within ~3 min, as confirmed by infrared (IR) spectroscopy (fig. S12). After diluting the reaction mixture with chloroform, we could visually discriminate the absolute configuration of **2a**, without isolating the modified polymers (movie S1). Thus, a practically useful dual-mode, on-site chiral sensor (visual differences in solution color and fluorescence emission) could be developed.

**Fig. 3:**
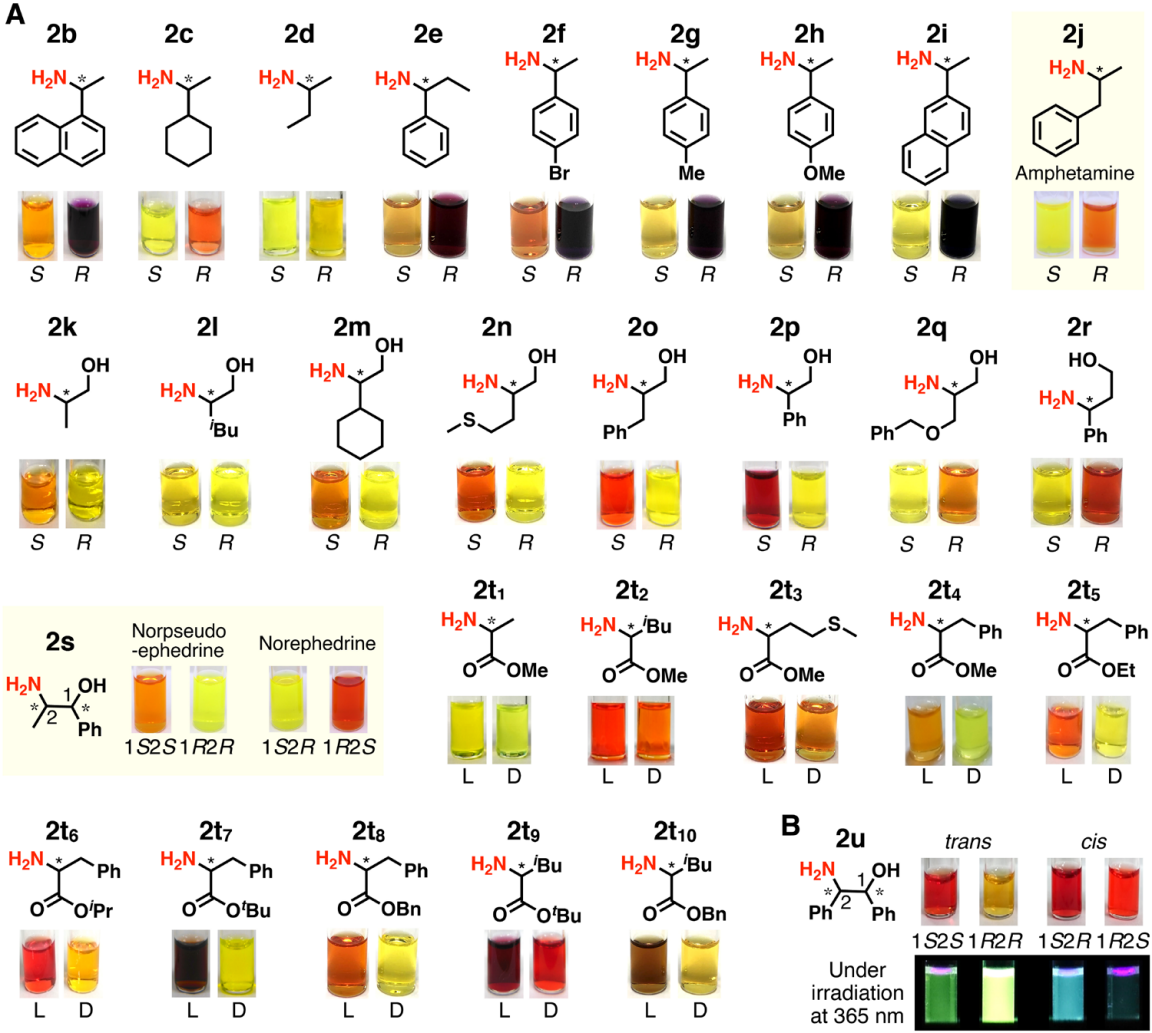
Naked-eye detection of enantiomers of various amines. (**A**) Color of *M*-*h*-poly-**1**-**2b**–**2t** (1.0 mM) in THF at 25°C (**2b**, **2f** to **2i**, **2p**, **2r**, **2t**_**7**_, and **2t**_**10**_) and −50°C (**2j**), in chloroform at 25°C (**2c**, **2q**, **2s**, **2t**_**5**_, and **2t**_**6**_) and −60°C (**2d** and **2t**_**1**_ to **2t**_**4**_), in THF-acetone (6/4, v/v) at 25°C (**2e**), in chloroform-methanol (5/1, v/v) at −50°C (**2k**), in chloroform-methanol (19/1, v/v) at −60°C (**2l**), in chloroform-methanol (8/2, v/v) at −60°C (**2n**), and in chloroform-THF (9/1, v/v) at 25°C (**2o**, **2t**_**8**_, and **2t**_**9**_) and −60°C (**2m**). (**B**) Color and fluorescent emission of *M*-*h*-poly-**1**-**2u** (1.0 or 0.01 mM) in THF at 25°C under white light (top) and upon irradiation at 365 nm (bottom).

It was possible to develop a similar assay for the naked-eye detection of chirality for various chiral amines (**2b** to **2j**), amino alcohols (**2k** to **2s**), and amino acid esters (**2t**_**1**_ to **2t**_**10**_) upon their reaction with *M*-*h*-poly-**1**-H (Fig. 3A), followed by solvent (fig. S13) and temperature (fig. S14) optimization. For simple chiral amine **2d**, amino alcohols **2k** to **2n**, and amino acid methyl esters **2t**_**1**_ to **2t**_**4**_, naked-eye detection was possible at a low temperature (−60° or −50°C). The colorimetric response from the amino acid esters notably improved by introducing bulkier ester groups, particularly, the *tert*-butyl and benzyl ester groups, allowing the naked-eye detection of the enantiomers at 25°C (**2t**_**7**_ to **2t**_**10**_) (Fig. 3A and fig. S13). All the tested primary amines (**2a** to **2j**), amino alcohols (**2k** to **2s**), and amino acid esters (**2t**_**1**_ to **2t**_**10**_) with the same configuration as *R***2a**, *S***2k**, and L**2t**_**1**_, respectively, exhibited more prominent color changes than their corresponding enantiomeric counterparts after functionalization with *M*-*h*-poly-**1**-H except for **2q** and **2r**, of which the (*R*)-enantiomers exhibited a more prominent color change because of difference in the priority sequences (see fig. S13B). This allowed the quick assignment of the chiral amine configurations by simple visible inspection. Enantiomers of representative drug-related compounds, such as amphetamine (**2j**)—a stimulant drug and a metabolite of other stimulant drugs, and phenylpropylamine (norpseudoephedrine and norephedrine) (**2s**), could also be visually discriminated (Fig. 3A and fig. S15). Their chiral discrimination is important because it is useful to distinguish the source of the drugs (*2*). Four diastereomers of **2u** with two stereogenic centers could also be discriminated by the naked eye and fluorescence emission upon irradiation at 365 nm (Fig. 3B and fig. S16). In sharp contrast, *M*-*h*-poly-**1**-*S***2a**^**Me**^ and *M*-*h*-poly-**1**-*R***2a**^**Me**^ composed of secondary chiral amines prepared from *N*-methylated **2a** (*S*- and *R*-**2a**^**Me**^) exhibited almost identical absorption and CD spectra (fig. S13A), suggesting that cooperative intramolecular H-bonding between the neighboring amide pendants along the helical backbone is crucial in the colorimetric chiral discrimination by this system and chiral secondary amines cannot be applied to this method.

**Fig. 4:**
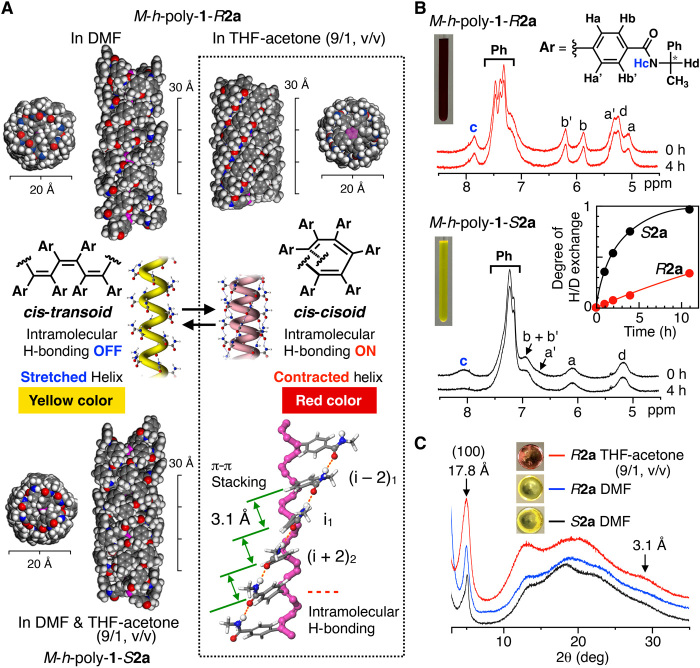
Structures of helical polymers relevant to colorimetric chiral recognition of amines. (**A**) Calculated left-handed helical structures of *cis-cisoidal M*-*h*-poly-**1**-*R***2a** (top right), *cis-transoidal M*-*h*-poly-**1**-*R***2a** (top left), and *M*-*h*-poly-**1**-*S***2a** (bottom left). *Cis-cisoidal M*-*h*-poly-**1**-*R***2a** forms an intramolecularly H-bonded network between the amide pendants and a π-π stacking of the phenyl pendants [i_1_ and (i + 2)_2_, i_1_ and (i – 2)_1_; bottom right]. (**B**) Time-dependent ^1^H NMR spectra of 2.5 mM *M*-*h*-poly-**1**-*R***2a** (top) and *M*-*h*-poly-**1**-*S***2a** (bottom) in THF-*d*_8_–acetone-*d*_6_ (9/1, v/v, 570 μl) after addition of CD_3_CO_2_D (30 μl) at 25°C. Insets show the plots of the degree of H/D exchange for the amide protons of *M*-*h*-poly-**1**-*R***2a** (red) and *M*-*h*-poly-**1**-*S***2a** (black) and photographs of the solutions at 25°C. (**C**) XRD patterns of *M*-*h*-poly-**1**-*R***2a** film prepared by casting THF-acetone (9/1, v/v) solution (red), XRD patterns of *M*-*h*-poly-**1**-*R***2a** (blue) and *M*-*h*-poly-**1**-*S***2a** (black) films prepared by casting DMF solution, and photographs of the films at 25°C.

Direct evidence for the difference between the intramolecular H-bonding among the adjacent amide pendants in red-colored *M*-*h*-poly-**1**-*R***2a** and yellow-colored *M*-*h*-poly-**1**-*S***2a** in THF-acetone (9/1, v/v) was obtained from amide hydrogen-deuterium (H/D) exchange experiments (Fig. 4B and fig. S21). The half-lives of the H/D exchange for the amide protons of *M*-*h*-poly-**1**-*R***2a** and *M*-*h*-poly-**1**-*S***2a**, as estimated from the Arrhenius analysis, are 19 and 2 hours, respectively, indicating that the intramolecular H-bonding in red-colored *M*-*h*-poly-**1**-*R***2a** was much stronger than in yellow-colored *M*-*h*-poly-**1**-*S***2a**; this was also supported by the IR measurements (fig. S22).

Molecular mechanics calculations revealed that *cis-cisoidal M*-*h*-poly-**1**-*R***2a** can form regular intramolecular H-bonds between the neighboring amide pendants, whereas *cis-cisoidal M*-*h*-poly-**1**-*S***2a** can only partially form these intramolecular H-bonds (Fig. 4A and fig. S23). Moreover, this regular intramolecular H-bonding is impossible in *cis-transoidal M*-*h*-poly-**1**-*R***2a** and *M*-*h*-poly-**1**-*S***2a** (Fig. 4A). Therefore, the behaviors of *M*-*h*-poly-**1**-*R***2a** and *M*-*h*-poly-**1**-*S***2a** can be ascribed to a spring-like helical conformational change in the contracted *cis-cisoidal* helical conformation (red-colored) and stretched *cis-transoidal* conformation (yellow-colored), respectively (*31*); the color change in the former is triggered by the formation of regular intramolecular H-bonding networks among the amide pendants in specific polar solvents, thus acting as an on/off switch. X-ray diffraction (XRD) patterns of red-colored *M*-*h*-poly-**1**-*R***2a** and yellow-colored *M*-*h*-poly-**1**-*R***2a** and *M*-*h*-poly-**1**-*S***2a** films showed a strong reflection at 17.8 Å, which can be indexed to (100) reflections, suggesting a columnar pseudohexagonal packing (Fig. 4C and fig. S24) (*31*). The red-colored *M*-*h*-poly-**1**-*R***2a** film showed a weak but apparent reflection at 28.5°, assigned to the π-π stacking of the pendant phenyl rings (3.1 Å). In contrast, the yellow-colored films showed no observable reflection in the same region. These results suggested that red-colored *M*-*h*-poly-**1**-*R***2a** forms π-π stacking of the pendant phenyl rings by adopting the contracted *cis-cisoidal* conformation through the formation of intramolecular H-bonds between the neighboring amide groups (Fig. 4A, right bottom); this π-π stacking is not available in the yellow-colored, stretched *cis-transoidal M*-*h*-poly-**1**-*R***2a** and *M*-*h*-poly-**1**-*S***2a**.

These results indicate that the degree of color change (difference) between the diastereomeric amide-bound one-handed helical polymers resulting from the formation or disruption (switching on/off) of the intramolecular H-bonding networks among the pendant amides is most likely determined by a delicate balance of the polarity and the intermolecular H-bonding ability (solvation) of the solvents with the chiral amide residues, temperature, and steric effect of the hydrophobic or hydrophilic substituents on the stereogenic center of the amide pendants derived from the enantiomers of primary amines. When one-handed helical *M*-*h*-poly-**1**-H was modified with chiral amines and amino alcohols bearing a bulky hydrophobic substituent [e.g., phenyl (**2a**, **2f** to **2h**, **2p**, and **2r**), naphthyl (**2b** and **2i**), cyclohexyl (**2c**), and benzyl (**2j** and **2o**)] over the other small substituents on the stereogenic center, the naked-eye detection of the enantiomers of the amines was possible at 25°C in polar THF and/or less polar chloroform solvents (Fig. 3A and figs. S13 and S15). This is because of the chiral steric effect of the bulky hydrophobic substituents on the stereogenic center, which further enables to suppress solvation of the amide residues in the solvents used. Therefore, one of the diastereomeric amide-bound one-handed helical polymers can form the intramolecular H-bonding networks in the solvents. On the other hand, when *M*-*h*-poly-**1**-H was modified with a chiral amine and amino alcohol carrying a less bulky ethyl (**2d**) and methyl (**2k**) substituent, respectively, on the stereogenic center, the naked-eye detection of the enantiomers of **2d** and **2k** required low-temperature measurements in less polar chloroform as anticipated (Fig. 3A) because of easy access of solvent molecules to the amide groups along with insufficient chiral steric effect, which will prevent the formation of the intramolecular H-bonds between the neighboring amide pendants at 25°C. As for the amino acid methyl esters (**2t**_**1**_ to **2t**_**4**_), the methyl ester residue is bulky but hydrophilic so that the amide residues are more easily solvated, although **2t**_**2**_ and **2t**_**4**_ have a bulky hydrophobic *iso*-butyl and benzyl substituent on the stereogenic center, respectively, requiring low-temperature measurements in less polar chloroform at −60°C (**2t**_**2**_) and −20°C (**2t**_**4**_) for the naked-eye detection of the enantiomers (Fig. 3A and fig. S14). Hence, introducing bulkier hydrophobic ester groups, such as the ethyl (**2t**_**5**_), isopropyl (**2t**_**6**_), *tert*-butyl (**2t**_**7**_), and benzyl (**2t**_**8**_) ester groups, strongly suppresses solvation of the amide residues, which enables to form the intramolecular H-bonding networks between the neighboring amide pendants along one of the diastereomeric helical polymer backbones, thus allowing the naked-eye detection of the enantiomers in chloroform, THF, or its mixture at 25°C (Fig. 3A). In polar DMF, visible color change was not observed for all the tested enantiomers of amines and the solutions remained yellow independent of the chirality (figs. S13 and S15).

**Fig. 5:**
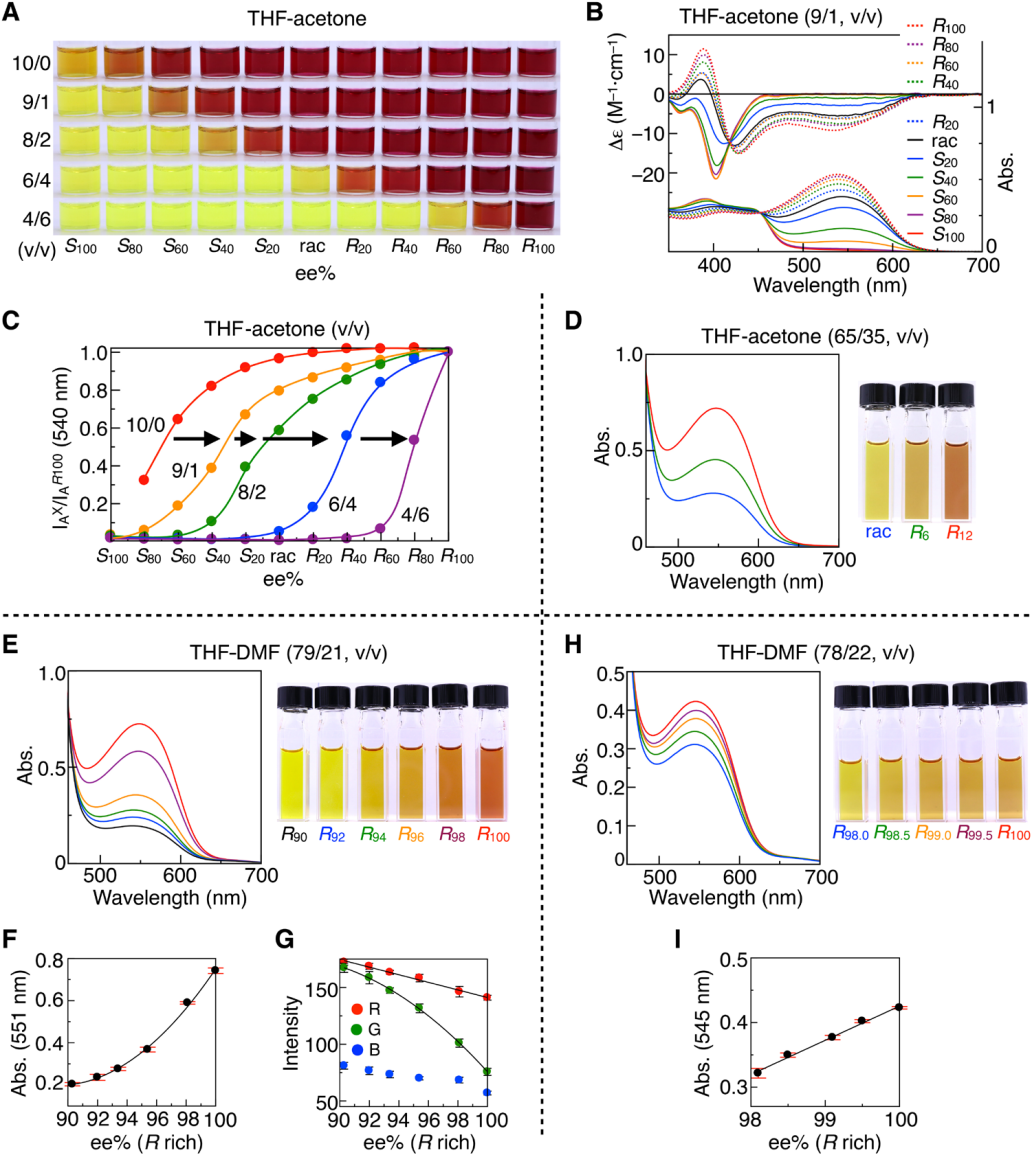
Quantitative determination of the full range of enantiomeric excess of chiral amine 2a. (**A**) Solvent-dependent color changes of 1.0 mM *M*-*h*-poly-**1**-**2a** in THF-acetone mixtures at 25°C. (**B**) CD and absorption spectra of 1.0 mM *M*-*h*-poly-**1**-**2a** in THF-acetone (9/1, v/v) at 25°C. (**C**) Plots of the relative absorption intensity (I_A_^X^/I_A_^*R*100^) of *M*-*h*-poly-**1**-**2a** at 540 nm in THF-acetone mixtures versus the pendant ee of **2a**. (**D**, **E**, and **H**) Absorption spectra and color of 0.5 mM *M*-*h*-poly-**1**-**2a** [pendant ee = 0, *R*_6_, *R*_12_% (D), *R*_90_ to *R*_100_, in steps of 2% (E), *R*_98_ to *R*_100_, in steps of 0.5% (H)] in THF-acetone (65/35, v/v) (D) and THF-DMF [79/21, v/v (E) and 78/22, v/v (H)] at 25°C. (**F** and **G**) Plots of the absorption intensity of *M*-*h*-poly-**1**-**2a** at 551 nm (F) and RGB values obtained by digital photography (G) in THF-DMF (79/21, v/v) versus the pendant ee of **2a**. (**I**) Plots of the absorption intensity of *M*-*h*-poly-**1**-**2a** at 545 nm in THF-DMF (78/22, v/v) versus the pendant ee of **2a**. Error bars represent the SD from three measurements. Photo credit: Mai Nozaki, Kanazawa University.

We then used our helical polymer–based color indicator (*M*-*h*-poly-**1**-H) for determining the ee of chiral amine **2a**. The method was based on the simple and quick functionalization of the pendant carboxy groups of *M*-*h*-poly-**1**-H with various proportions of **2a**, ranging from 100% *S*-**2a** (*S*_100_-**2a**) to 100% *R*-**2a** (*R*_100_-**2a**), in steps of 20% ee. The absorption spectra of *M*-*h*-poly-**1**-**2a** in THF-acetone (9/1, v/v) red-shifted nonlinearly with increasing *R*-**2a** content, with a clear isosbestic point at 465 nm (Fig. 5, B and C); the solution color changed from yellow to red at around *S*60 (Fig. 5A). A similar nonlinear response toward ee of **2a** was also observed in the fluorescence spectra of *M*-*h*-poly-**1**-**2a** (figs. S25A and S26). As anticipated, upon further addition of acetone (table S1), the ee-dependent range for visible color change shifted to the *R*-rich side (Fig. 5, A and C, and fig. S27), allowing approximate determination of the ee values of **2a** by visual inspection (Fig. 5A) and quantitative determination of the ee from the absorption spectral changes (Fig. 5C). These color changes arise most likely due to a spring-like conformational change in *M*-*h*-poly-**1**-**2a** from the stretched *cis-transoidal* structure (yellow) to the contracted *cis-cisoidal* structure (red). The conformational change is regulated by polar solvents (acetone in this case), which affect the intramolecular H-bonds as discussed above. *M*-*h*-poly-**1**-**2a** also exhibited a similar ee-dependent visible color change in the presence of an increasing amount of aprotic polar solvents, such as methyl isobutyl ketone (MIK), DMF, dimethylacetamide (DMA), and dimethyl sulfoxide (DMSO) in THF (fig. S28); polar solvents with higher dielectric constants (ε; table S1) showed a larger shift upon the addition of a small amount of the polar solvent (*32*). Similarly, naked-eye detection of the configuration and ee values of other chiral amines (**2b**, **2c**, **2p**, and **2t**_**7**_) is possible after simple and quick functionalization of the amines with *M*-*h*-poly-**1**, followed by their dissolution in appropriate solvent mixtures (figs. S29 and S30).

Taking advantage of the unique ee-dependent color changes of *h*-poly-**1**-**2a** regulated by the solvent polarity, more precise determination of the ee of **2a**, based on the color change, was conducted in various narrow ee regions. For example, *M*-*h*-poly-**1**-*R*_X_**2a** [*X* (% ee) = 0, 6, 12] could be clearly distinguished by naked eyes using a THF-acetone mixed solvent (65/35, v/v; Fig. 5D and fig. S31). Moreover, *M*-*h*-poly-**1**-*R*_X_**2a** [*X* (% ee) = 90 to 100, in steps of 2] prepared by the reaction of *M*-*h*-poly-**1**-H with the corresponding chiral amines *R*_X_-**2a** (table S3) showed substantial changes in the absorption intensity at 551 nm and apparent visible color changes with respect to the ee values of **2a** in THF-DMF (79/21, v/v). This allowed quantification of the difference in the ee values with high accuracy (Fig. 5, E and F). Naturally, when *P*-*h*-poly-**1**-H, with opposite helicity memory, was modified with *S*_X_-**2a** [*X* (% ee) = 90 to 100, in steps of 2], the *P*-*h*-poly-**1**-*S*_X_**2a** enantiomers (table S3) displayed absorption spectral changes and color changes (fig. S32) similar to those of *M*-*h*-poly-**1**-*R*_X_**2a**. It is also to be noted that optically active *M*- and *P*-*h*-poly-**1**-H compounds reacted with nonracemic **2a** in a nonselective way (table S4). The ee difference between *R*_90_ and *R*_100_ of **2a** could also be visually discriminated by their fluorescence (fig. S33).

The ee values of “unknown” **2a** samples, ranging from *R*_90_ to *R*_100_, were estimated from their absorption spectra based on the calibration curve. The values were very close to the ee values determined by chiral HPLC, and the errors were relatively low (fig. S34 and table S5) (*16*, *33*). Furthermore, colorimetric determination of ee without using any spectroscopic instruments was possible by taking photographs of the solutions and converting them to RGB values (fig. S34D). Hence, we could estimate the ee values of *R*_X_-**2a** (*X* ≥ 90) with high accuracy from the plots of the intensities of the G (green) component (Fig. 5G and table S5).

To investigate whether this system can detect an extremely small difference in the ee in a sample with very high ee of **2a** (≥98), *M*-*h*-poly-**1**-*R*_X_**2a** [*X* (% ee) = 98 to 100, in steps of 0.5] was also prepared (table S3). Apparent absorption spectral changes were observed for *M*-*h*-poly-**1**-*R*_X_**2a** (*X* ≥ 98) in THF-DMF (78/22, v/v) (Fig. 5H); the plots of their absorbance at 545 nm were linear with respect to the ee values of **2a** (Fig. 5I). The *P*-*h*-poly-**1**-*S*_X_**2a** enantiomers with the opposite helicity memory (table S3) showed identical absorption spectral changes (fig. S35). These results demonstrated that using this unique colorimetric sensor, a difference in the ee values as small as 0.5% ee, even in a sample with a very high ee (≥98), could be detected by acquiring the absorption spectra.

We envisage that the pendant carboxy groups of *h*-poly-**1**-H can be replaced with various other functional groups while maintaining its macromolecular helicity memory. This should be applicable to the on-site, naked-eye determination of ee of various functional molecules and biologically relevant compounds.

## MATERIALS AND METHODS

### Materials

Tungsten(VI) chloride (WCl_6_) and (trimethylsilyl)diazomethane (TMSD; 2 M in diethyl ether) were purchased from Sigma-Aldrich (St. Louis, MO, USA). Tetraphenyltin (Ph_4_Sn) and L-(−)- and D-(+)-mandelic acids were obtained from Tokyo Chemical Industry (TCI; Tokyo, Japan). Potassium hydroxide (KOH) and 2-methoxy-4-nitrobenzoic acid were purchased from FUJIFILM Wako Pure Chemical (Osaka, Japan). Optically active amines (**2a** to **2i**, **2k** to **2r**, **2t**_**1**_ to **2t**_**10**_, **2u**, and **2a**^**Me**^) (Fig. 3 and fig. S3) were obtained from Sigma-Aldrich, FUJIFILM Wako Pure Chemical, TCI, Nacalai Tesque (Kyoto, Japan), and Watanabe Chemical Industries (Hiroshima, Japan) and used as received except for **2a** (see the Supplementary Materials). (1*S*,2*R*)- and (1*R*,2*S*)-norephedrine (1*S*2*R*- and 1*R*2*S*-**2s**) were purchased from FUJIFILM Wako Pure Chemical. (*S*)- and (*R*)-amphetamine (*S*- and *R*-**2j**) (*34*) and (1*S*,2*S*)- and (1*R*,2*R*)-norpseudoephedrine (1*S*2*S*- and 1*R*2*R*-**2s**) (*35*) were synthesized from the corresponding norephedrine according to the literature method. Anhydrous THF, toluene, DMSO, and DMF were purchased from Kanto Kagaku (Tokyo, Japan), and these solvents were stored under nitrogen. Dehydrated acetone and MIK were obtained from FUJIFILM Wako Pure Chemical. DMT-MM was prepared according to the reported method (*36*). Poly-**1**-H was prepared according to the previously reported method (see the Supplementary Materials) (*28*, *29*).

### Instruments

Nuclear magnetic resonance (NMR) spectra were taken on a JNM-ECA 500 spectrometer (500 MHz for ^1^H, 125 MHz for ^13^C) (JEOL, Akishima, Japan) or a Bruker Avance 400 spectrometer (400 MHz for ^1^H, 100 MHz for ^13^C) (Bruker, MA, USA) in CDCl_3_, DMSO-*d*_6_, DMF-*d*_7_, CD_3_OD, THF-*d*_8_, THF-*d*_8_–acetone-*d*_6_ (9/1, v/v), and THF-*d*_8_–CDCl_3_ (5/1, v/v) using TMS (for CDCl_3_, THF-*d*_8_, THF-*d*_8_–acetone-*d*_6_, and THF-*d*_8_–CDCl_3_, ^1^H and ^13^C) or a solvent residual peak (for DMSO-*d*_6_, DMF-*d*_7_, and CD_3_OD, ^1^H and ^13^C) as the internal standard. IR spectra were recorded with a JASCO Fourier Transform IR-460 spectrophotometer (JASCO, Tokyo, Japan). Absorption and CD spectra were measured in a 1.0-mm, 10-mm, or 0.1-mm quartz cell on a JASCO V-650 spectrophotometer and a JASCO J-725 spectropolarimeter, respectively. The temperature was controlled with a JASCO PTC-348WI apparatus. The concentration of polymers was calculated on the basis of the monomer units. Size exclusion chromatography (SEC) measurements were performed with a JASCO PU-2080 liquid chromatograph equipped with an ultraviolet (UV)–vis (JASCO UV-970) detector at 40°C using a Shodex (Tokyo, Japan) KF-805L SEC column. The temperature was controlled with a JASCO CO-1560 column oven. THF was used as the eluent at a flow rate of 1.0 ml/min. The molar mass calibration curves were obtained with polystyrene standards (Tosoh, Tokyo, Japan). Photoluminescence spectra were measured on a JASCO FP-8500 spectrofluorometer. The temperature was controlled with a JASCO ETC-815 apparatus. XRD measurements were performed on Nano Viewer (RA-MICRO7HFM) (Rigaku Corporation, Tokyo, Japan). Ee values of chiral amines (**2a** to **2c**, **2p**, and **2t**_**7**_) were determined by HPLC equipped with a photodiode array detector (JASCO MD-4010) and a CD detector (JASCO CD-4095) at room temperature (see the Supplementary Materials). A chiral column [CHIRALPAK IG-3; 250 × 4.6 mm inner diameter (i.d.); Daicel, Osaka, Japan] was connected, and *n*-hexane–dichloromethane (1/1, v/v) was used as the eluent at a flow rate of 0.5 ml/min. A chiral column [CROWNPAK CR-I (−); 250 × 4.6 mm i.d.; Daicel] was also used for ee determination of **2t**_**7**_·HCl. pH 1.5 HClO_4_ aq./CH_3_CN (4/1, v/v) was used as the eluent at a flow rate of 0.4 ml/min. Elemental analyses were performed by the Research Institute for Instrumental Analysis of Advanced Science Research Center, Kanazawa University, Kanazawa, Japan.

### Synthesis

#### General procedure for the modification of M- and P-h-poly-1-Hs with chiral amines

The reactions of *M*-*h*-poly-**1**-H or *P*-*h*-poly-**1**-H with chiral amines (**2a** to **2u** and **2a**^**Me**^) (Fig. 3 and fig. S3) were carried out with DMT-MM as the condensing reagent, as shown in fig. S4. A typical experimental procedure for the reaction of *M*-*h*-poly-**1**-H with chiral amine **2a** is described below.

*S*-**2a** (95.7 μl, 0.75 mmol) and DMT-MM (209 mg, 0.75 mmol) were added to a solution of *M*-*h*-poly-**1**-H (50.4 mg, 0.19 mmol) in a DMSO-water mixed solvent (5/1, v/v) (10 ml), and the resulting mixture was stirred at room temperature for 4 hours. The resulting polymer was precipitated into a large amount of methanol-water mixture (1/1, v/v), collected by centrifugation, washed with methanol-water mixture (1/1, v/v), and then dried in vacuo at room temperature overnight to yield *M*-*h*-poly-**1**-*S***2a** (85.0 mg, 0.18 mmol, 95% yield). The side groups of *M*-*h*-poly-**1**-H were completely modified with *S*-**2a** as confirmed by its ^1^H NMR and elemental analysis.

In the same way, modifications of *M*-*h*-poly-**1**-H with *R*-**2a** and other various *R* and *S* amines (**2b** to **2u** and **2a**^**Me**^) were performed to afford the corresponding *M*-*h*-poly-**1**-*R***2**s and *M*-*h*-poly-**1**-*S***2**s. The complete modifications of the side groups were confirmed by ^1^H NMR, IR, and elemental analyses (see the Supplementary Materials and also table S6 and fig. S12).

#### Procedure for the absorption spectral measurements of M-h-poly-1-R_X_2a and P-h-poly-1-S_X_2a (X > 90% ee)

Stock solutions of *M*-*h*-poly-**1**-*R*_X_**2a** (7.5 mM) (*X* = 90, 92, 94, 96, 98, and 100% ee) in THF were prepared in 2-ml flasks equipped with a stopcock. A 200-μl aliquot of each *M*-*h*-poly-**1**-*R*_X_**2a** stock solution was transferred to three vials using a Hamilton microsyringe. THF was completely removed under a high vacuum to give three vials containing 1.5 μmol of *M*-*h*-poly-**1**-*R*_X_**2a**. A mixed solvent THF-DMF (79/21, v/v) was prepared by mixing THF (140.30 g) and DMF (39.69 g) in a bottle with a Teflon screw cap. A 3-ml aliquot of the mixed solvent was added to the vials to keep the *M*-*h*-poly-**1**-*R*_X_**2a** concentrations at 0.5 mM. The absorption spectra were taken at 25°C using a 10-mm quartz cell for each vial, and the isosbestic point was observed at 456 nm. The concentration of the polymers was corrected using the ε (molar absorptivity) value (ε_456_ = 2.0 × 10^3^ M^−1^·cm^−1^). The average absorption intensity at 551 nm of each *M*-*h*-poly-**1**-*R*_X_**2a** sample was determined on the basis of the results of three vials. The average absorption intensities at 551 nm were plotted versus the % ee values of the samples determined by the chiral HPLC analysis (tables S2 and S3 and fig. S32C). This relationship was used as the calibration curve for ee determination of blind unknown *M*-*h*-poly-**1**-*R*_X_**2a** (*X* > 90) samples using absorption intensities at 551 nm (fig. S34B) (see below). The same procedure was used for the absorption spectral measurements of *P*-*h*-poly-**1**-*S*_X_**2a** (*X* = 90, 92, 94, 96, 98, and 100% ee).

The absorption spectral measurements of *M*-*h*-poly-**1**-*R*_X_**2a** and *P*-*h*-poly-**1**-*S*_X_**2a** (*X* = 98.0, 98.5, 99.0, 99.5, and 100% ee) were carried out in a similar way by using a mixed solvent THF-DMF (78/22, v/v), which was prepared by mixing THF (138.53 g) and DMF (41.58 g) in a bottle with a Teflon screw cap. The absorption spectra were taken at 25°C using a 10-mm quartz cell for each vial, and the isosbestic point was observed at 456 nm. The concentration of the polymers was corrected using the ε (molar absorptivity) value (ε_456_ = 2.0 × 10^3^ M^−1^·cm^−1^). The average absorption intensity at 545 nm of each *M*-*h*-poly-**1**-*R*_X_**2a** sample was determined on the basis of the results of three vials. The average absorption intensities at 545 nm were plotted versus the % ee values of the samples determined by the chiral HPLC analysis (tables S2 and S3 and fig. S35D).

#### Procedure of the determination of the ee values of blind unknown R_X_-2a samples

##### Preparation of blind unknown R_X_-2a for modification of M-h-poly-1-H

Two blind unknown *R*_X_-**2a** samples [sample A (*R*_X_-**2a**A) and sample B (*R*_X_-**2a**B)] were prepared by mixing *R*_90_-**2a** and *R*_100_-**2a** at random with a Hamilton microsyringe. The ee values of *R*_X_-**2a**A and *R*_X_-**2a**B were determined to be 92.9% and 96.8% ee by chiral HPLC analysis (table S5) after derivatization with 2-methoxy-4-nitrobenzoic acid into the corresponding amide compound **3a** in the same way as described in the Supplementary Materials (see section S3-1). The modification of *M*-*h*-poly-**1**-H with *R*_X_-**2a**A and *R*_X_-**2a**B was performed in the same way as described above [THF-water mixed solvent (4/1, v/v) was used instead of DMSO-water mixed solvent] to afford *M*-*h*-poly-**1**-*R*_X_**2a**A and *M*-*h*-poly-**1**-*R*_X_**2a**B.

##### Procedure of the determination of the ee values of blind unknown R_X_-2a samples after converting to M-h-poly-1-R_X_2a based on their absorption spectra

Stock solutions of *M*-*h*-poly-**1**-*R*_X_**2a**A and *M*-*h*-poly-**1**-*R*_X_**2a**B (7.5 mM) in THF were prepared in 2-ml flasks equipped with a stopcock. A 200-μl aliquot of each *M*-*h*-poly-**1**-*R*_X_**2a**A and *M*-*h*-poly-**1**-*R*_X_**2a**B stock solution was transferred to three vials using a Hamilton microsyringe. THF was completely removed under a high vacuum to give three vials containing 1.5 μmol of *M*-*h*-poly-**1**-*R*_X_**2a**A and *M*-*h*-poly-**1**-*R*_X_**2a**B. A mixed solvent THF-DMF (79/21, v/v) was prepared by mixing THF (140.30 g) and DMF (39.69 g) in a bottle with a Teflon screw cap. A 3-ml aliquot of the mixed solvent was added to the vials to keep the *M*-*h*-poly-**1**-*R*_X_**2a**A and *M*-*h*-poly-**1**-*R*_X_**2a**B concentrations at 0.5 mM. The absorption spectra were taken at 25°C using a 10-mm quartz cell for each vial, and the isosbestic point was observed at 456 nm (fig. S34A). The concentration of the polymers was corrected using the ε (molar absorptivity) value (ε_456_ = 2.0 × 10^3^ M^−1^·cm^−1^). The average absorption intensity at 551 nm of each *M*-*h*-poly-**1**-*R*_X_**2a**A and *M*-*h*-poly-**1**-*R*_X_**2a**B sample was determined on the basis of the results of three vials (table S5). The calibration curve was obtained from the relationship between the absorption intensities at 551 nm and the ee % values of the *M*-*h*-poly-**1**-*R*_X_**2a** (*X* = 90, 92, 94, 96, 98, and 100) samples (fig. S34B) (see above). By using this calibration curve, the % ee values of *R*_X_-**2a**A and *R*_X_-**2a**B were estimated to be 92.5% and 96.3% ee, respectively (fig. S34B and table S5).

##### Procedure of the determination of the ee values of blind unknown R_X_-2a samples by taking their photographs

Photographs of the solutions in 10-mm quartz cells of *M*-*h*-poly-**1**-*R***2a**A, *M*-*h*-poly-**1**-*R***2a**B, and *M*-*h*-poly-**1**-*R*_X_**2a** (*X* = 90, 92, 94, 96, 98, and 100) samples were taken at 25°C under white light using a digital camera (CANON EOS Kiss M). Color values as RGB of the photo images of the solutions displayed on the screen were detected by using the Digital Color Meter (Apple Inc.), which is a built-in Mac utility. Ten spots of the photo images were randomly selected, and the color values as RGB of the solutions were determined by averaging them of the 10 spots. The calibration curve was obtained from the relationship between the G (green) values and the ee % values of the *M*-*h*-poly-**1**-*R*_X_**2a** (*X* = 90, 92, 94, 96, 98, and 100) samples (fig. S34C). By using this calibration curve, the ee % values of the blind samples (*M*-*h*-poly-**1**-*R***2a**A and *M*-*h*-poly-**1**-*R***2a**B) were determined from the G values of the blind samples (table S5). Solution samples for calibration curves were prepared on the same day to minimize errors during the measurements.

## SUPPLEMENTARY MATERIALS

Supplementary material for this article is available at http://advances.sciencemag.org/cgi/content/full/7/27/eabg5381/DC1
